# Do Genes Respond to Global Warming?

**DOI:** 10.1371/journal.pbio.0020338

**Published:** 2004-09-07

Scientists continue to argue the extent that human activities drive global warming, but few would argue that it exists. The International Panel on Climate Change predicts that greenhouse gases will increase global temperatures by 3.6 degrees F by 2100—a rise unprecedented over the past 10,000 years. What might the world look like as we approach that point? Wetlands will disappear. Floods, hurricanes, and droughts will become progressively more severe. Infectious diseases will increase in virulence and range. Montana's famed glaciers may all but disappear within 30 years. A quarter of species may vanish by 2050.

While the effects of climate change on species' geographic ranges and population dynamics have been studied to some extent, scientists know little about how species respond to climate change at the genetic level. In this issue, Elizabeth Hadly and colleagues analyze three different dynamic processes—environmental change, population response, and gene diversity fluctuations—and present evidence that climate change influences variation in genetic diversity.

Focusing on two mammal species—the Montane vole and the northern pocket gopher—Hadly et al. asked how the two species responded to historical climate-induced habitat alterations in northwestern Wyoming. They gathered fossils from Yellowstone National Park's Lamar Cave, which contains a treasure trove of carbon-dated deposits that mirror the community of mammals in the area today. Comparing genetic material extracted from fossil samples from different time points over the past 3,000 years to genetic data taken from contemporary animals, Hadly's team tracked genetic changes in populations of the two species and used this information (along with relative fossil abundance and modern population density) to estimate changes in effective population size over time. (Effective population size refers to the number of individuals contributing genetic material to the next generation. Populations with a small effective population size, for example, would be highly vulnerable to environmental catastrophe.) The genetic and demographic data were then combined with environmental records to analyze the relationship between the factors.

Studying these populations in space and time—an approach the authors call “phylochronology”—offers an opportunity to analyze the genetic diversity of a species against the backdrop of environmental fluctuation within an evolutionary time frame. It also suggests how microevolutionary forces—factors that affect genetic variation in populations over successive generations—shape genetic responses to climate change. Such evolutionary forces include mutation, genetic drift (the random gene fluctuation in small populations that stems from the vagaries of survival and reproduction), and gene flow (changes in the gene frequency of a population caused by migration).[Fig pbio-0020338-g001]

**Figure pbio-0020338-g001:**
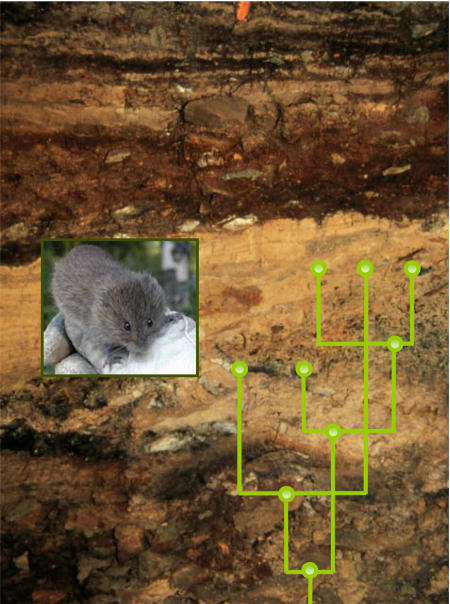


The past 3,000 years includes two periods marked by dramatic climate change—the Medieval Warm Period and the Little Ice Age—that had different effects on local mammal populations depending on their habitat preferences. Habitat specialists, the vole and pocket gopher live in the wet mountain regions of western North America. Though both showed population increases during wetter climates and declines during warmer periods, Hadly et al. predicted the gene diversity fluctuations of the two species would differ based on their different ecological behaviors. And that's what they found: genetic response is tied to population size. Pocket gophers have low population densities, stick close to home, and are fiercely territorial, while voles live in high-density populations and range more widely. For the gophers, population declines resulted in reduced gene diversity; for the voles—which have a larger effective population size and greater dispersal between populations—population declines resulted in increased gene diversity. But what forces underlie these differences in genetic variation?

A recent study suggests that migration (a primary agent of gene flow) is most common in and between low-density patches in vole populations, which implicates gene flow as the driver of gene diversity patterns. But the authors don't rule out selection as a possibility, and suggest how to go about resolving the question. Hadly et al. show that phylochronology opens a unique window onto the relatively recent evolutionary past and offers “the potential to separate cause from effect.” They also conclude that “differences in species demography can produce differential genetic response to climate change, even when ecological response is similar.” With a 3-degree temperature increase in just the past 50 years in the American West, conservation of biodiversity may well depend on such insights.

